# The Carry-Over Effect of Competition in Task-Sharing: Evidence from the Joint Simon Task

**DOI:** 10.1371/journal.pone.0097991

**Published:** 2014-06-04

**Authors:** Cristina Iani, Filomena Anelli, Roberto Nicoletti, Sandro Rubichi

**Affiliations:** 1 Department of Communication and Economics, University of Modena and Reggio Emilia, Reggio Emilia, Italy; 2 Department of Philosophy and Communication, University of Bologna, Bologna, Italy; 3 Department of Psychology, University of Bologna, Bologna, Italy; Goldsmiths, University of London, United Kingdom

## Abstract

The Simon effect, that is the advantage of the spatial correspondence between stimulus and response locations when stimulus location is a task-irrelevant dimension, occurs even when the task is performed together by two participants, each performing a go/no-go task. Previous studies showed that this joint Simon effect, considered by some authors as a measure of self-other integration, does not emerge when during task performance co-actors are required to compete. The present study investigated whether and for how long competition experienced during joint performance of one task can affect performance in a following joint Simon task. In two experiments, we required pairs of participants to perform together a joint Simon task, before and after jointly performing together an unrelated non-spatial task (the Eriksen flanker task). In Experiment 1, participants always performed the joint Simon task under neutral instructions, before and after performing the joint flanker task in which they were explicitly required either to cooperate with (i.e., cooperative condition) or to compete against a co-actor (i.e., competitive condition). In Experiment 2, they were required to compete during the joint flanker task and to cooperate during the subsequent joint Simon task. Competition experienced in one task affected the way the subsequent joint task was performed, as revealed by the lack of the joint Simon effect, even though, during the Simon task participants were not required to compete (Experiment 1). However, prior competition no longer affected subsequent performance if a new goal that created positive interdependence between the two agents was introduced (Experiment 2). These results suggest that the emergence of the joint Simon effect is significantly influenced by how the goals of the co-acting individuals are related, with the effect of competition extending beyond the specific competitive setting and affecting subsequent interactions.

## Introduction

In recent years there has been a growing interest in the study of how performance on a task might be influenced by the presence of other individuals concurrently performing the same or a different task. Increasing evidence indicates that during joint performance, planning and execution of the individual’s actions are influenced by the co-actor’s actions, even when there is no need or requirement to consider them. A demonstration of this influence has been provided by Sebanz, Knoblich, and Prinz [1] by means of a modified version of a well-known experimental paradigm used to assess response conflict in individual settings, the Simon task (e.g., [2]; see also [3]–[6]; see [7], [8] for reviews).

In Sebanz et al.’s [1] study, participants were required to respond to the color (red or green) of a ring appearing on a finger pointing to the left or to the right. When the task was performed by a single participant in charge of responding to both colors (two-choice condition), responses were faster and more accurate when the finger pointed in the same direction as the response signaled by the ring’s color than when it pointed in the opposite direction. This difference, known as the Simon effect, is thought to reflect a conflict, emerging at the response selection stage [6] between two alternative response codes, one generated on the basis of task instructions and the other automatically activated by stimulus spatial features (in Sebanz et al’ s study, the finger’s pointing direction). When the two responses correspond, no competition between response codes arises and a response can be easily executed. Instead, when the two responses do not correspond, the incorrect response needs to be aborted thus slowing down response times and increasing the number of errors.

Crucially, Sebanz et al. [1] showed that a similar effect (from now on, joint Simon effect) occurred even when participants performed the task in a complementary way, each being responsible for only one response (joint go/no-go condition). The importance of this finding can be better understood if one considers that the Simon effect typically does not emerge when a participant performs the same go/no-go task alone, without the co-actor, responding to only one stimulus color (individual go/no-go condition). In this latter case, only one response code is formed and hence no conflict between spatial codes occurs. The observation of a Simon effect in the joint go/no-go condition has been taken as evidence that, even though each participant was responsible for only half of the task and hence for only one response alternative, they represented their co-actor’s task and integrated their own and the other’s action alternatives in action planning, thus allowing the conflict between response codes to emerge (see [9] for a review; see also [10] for a critical review; see [11], [12] for a “non-social” interpretation of the joint Simon effect). The representation of co-actor’s task is supposed to be at the basis of joint action since it allows individuals, while performing a task together, to understand and to predict the other’s actions, and to coordinate with each other [13].

Iani, Anelli, Nicoletti, Arcuri, and Rubichi [14] recently reported that the joint Simon effect did not emerge when, during joint task performance, participants were required to compete one against the other (but see [15] for different results). In Iani et al.’s Experiment 2, participants were randomly paired and asked to perform the Simon task together, each responding to one stimulus color. Under the cooperative condition, participants were told that the pair with the fastest and most accurate responses would receive an economic reward. This condition allowed the emergence of a positive interdependence, as the success of one individual rendered the success of the other more likely. Under the competitive condition, they were told that the participant of the pair with the fastest and most accurate responses would receive an economic reward. This condition allowed the emergence of a negative interdependence, as the success of one member rendered the success of the other less likely. A joint Simon effect was found only when individuals were required to cooperate but not when they were required to compete. Since perceived positive interdependence is thought to be the precondition for group formation and for ingroup–outgroup differentiation [16], the authors argued that when two individuals are required to cooperate, they perceive themselves as part of the same social group, and as a consequence they may have a stronger tendency to integrate their respective actions in a common representation. On the contrary, when the other represents an obstacle toward goal attainment, as occurs in explicit competitive situations, he/she is more likely perceived as an outgroup member and individuals may be less motivated to coordinate their efforts and to be influenced by the other’s actions, this blocking the integration of self and other’s action into a shared representation. This view is in line with studies showing that interdependence may strongly influence how we perceive others, with competitive contexts increasing perceived intergroup and interpersonal differences (e.g., [17]) and involving less self-other merging (e.g., [18], [19]) as compared to cooperative contexts.

To note, social psychology studies suggest that the effects of competition may not be limited to the situation in which competition was experienced, but may rather be long-lasting and may affect the way others are perceived and represented at the cognitive level even when competition is no longer required. Relevant to this issue are the results by Sassenberg, Moskowitz, Jacoby, and Hansen [20] showing that competing or even thinking about a competition in an intergroup context might have “carry-over effects”. To explain how this occurs, they referred to the notion of mindset which has been widely used to explain the carry-over effects found in priming experiments (see [21] for a review) and which indicates a set of cognitive procedures activated to perform a task and to achieve a specific goal (e.g., [22]). According to Sassenberg et al. [20], the competitive mindset activated in one situation may be applied to unrelated situations that follow the original competition, working as a prime and affecting the perception of subsequent intergroup situations.

Crucially, Sherif and colleagues [23] showed that in an intergroup context the negative effects of competition lasted until superordinate goals, that is, goals that could be achieved only through full cooperation of the members of the different groups, became relevant. In their study, eleven- and twelve-years old children in a summer camp program were divided into two separate groups and involved in competitive activities in which only one group could win, hence precluding the success of the other one. While competition continued, hostility between the groups increased and it decreased only with the introduction of common goals that required them to cooperate.

Given this empirical evidence deriving from both cognitive and social psychology, in the present work, we aimed at assessing whether the carry-over effect of a competitive mindset is a basic phenomenon that takes place not only in an intergroup context, but even in dyadic joint action contexts characterized by less complex interactions between individuals. Specifically we investigated whether competition experienced during joint performance of one task can affect the way a subsequent and different joint task is performed. To this aim, in Experiment 1 participants were required to perform a joint Simon task before and after jointly performing a letter version of the Eriksen flanker task [24] in which they were required to either collaborate with or to compete against their co-agent. The joint flanker task was chosen for two main reasons: first, because it can be performed jointly by two co-actors; second, because there are numerous studies showing that both the individual and joint Simon effects are influenced by previous performance on spatial tasks characterized by stimulus-response links that favor a specific spatial response (e.g., [25]–[30]). In the letter version of the Eriksen flanker task the links between stimuli and responses are arbitrary and are not spatial in nature. In this way, we could avoid transfer effects on the Simon task that may be due to specific stimulus-response associations acquired during previous performance of the flanker task.

In the joint flanker task we manipulated whether the goals of the two participants were positively related (i.e., the success of one individual rendered also the success of the other more likely) and participants were required to cooperate to achieve these goals or whether they were negatively related (i.e., the success of one individual rendered the success of the other less likely) and participants were required to compete one against the other to achieve a personal goal. In Experiment 1, the joint Simon task was always performed under neutral instructions. This allowed us to assess whether the need to compete during the joint flanker task influenced the way the following joint Simon task was performed, either by reducing or eliminating the joint Simon effect. To investigate whether the requirement to cooperate overrides the carry-over effect of competition, in Experiment 2 participants were explicitly required to compete during the joint flanker task and to cooperate during the subsequent joint Simon task.

At the end of both experiments, participants were asked to judge on a 7-point bipolar differential semantic scale whether they found the experimental situation easy vs. difficult, pleasant vs. unpleasant, positive vs. negative, cooperative vs. competitive. This was done for two main reasons. First, we wanted to exclude the possibility that differences in performance could be due to emotional factors. There is indeed evidence that the joint Simon effect is affected by the mood state of the participants [31] and by the valence (positive vs. negative) of the interaction [32]. Second, we wanted to assess the effectiveness of the cooperative/competitive instruction manipulation. For the manipulation to be effective, participants instructed to outdo one another during the flanker task should perceive the task situation as more competitive than participants who are required to cooperate.

## Experiment 1

The aim of the present experiment was to assess whether performing a task under either cooperative or competitive instructions affects the way a following joint task is performed. To this end, paired participants were required to perform a joint Simon task with neutral instructions (that is, instructions that did not explicitly require them to cooperate or compete) before and after performing a joint flanker task with either cooperative or competitive instructions.

### Methods

#### Ethics statement

The study was conducted in accordance with the Declaration of Helsinki and all procedures were approved by the Department of Communication and Economics of the University of Modena and Reggio Emilia.

#### Participants

Thirty-two undergraduate students from the University of Modena and Reggio Emilia (25 females, age range: 20–32 years) took part in the experiment for course credit. All participants gave their written informed consent to participate in the study. All participants were right-handed and reported normal or corrected-to-normal vision. All were naive with regard to the hypotheses of the experiment and were debriefed about the experimental aims at the end of the experiment.

Once recruited, participants were randomly paired and each pair was randomly assigned to one of two experimental conditions (i.e., cooperative vs. competitive instructions).

#### Apparatus and stimuli

During the experiment, participants sat side-by-side in front of a 17-inch color monitor at a distance of about 60 cm. E-Prime 2.0 software was used for stimulus presentation and response collection.

Stimuli in the joint Simon task were red or green solid squares (2×2 cm), presented 4.5 cm to the left or to the right of a central fixation cross (1×1 cm). Stimuli in the joint flanker task were arrays of five letters (2.5×1 cm) presented in the center of the screen. The letters H, K, S, and C could serve as target; the letters H, K, S, C, and U could serve as flankers. In both tasks, responses were executed by pressing the “z” or “-” key of a standard Italian keyboard with the left or right index finger, respectively.

#### Procedure

The experiment consisted of three consecutive sessions, each separated by a 5-min interval. The whole experiment lasted about 45 minutes.

During the first and third sessions, participants performed a joint Simon task, whereas in the second session they performed the joint version of the Eriksen flanker task developed by Atmaca, Sebanz, and Knoblich [33]. Both Simon and flanker tasks were carried out jointly with participants sitting side-by-side in front of the same computer screen. Seating position was kept constant across the different sessions.

During the joint Simon task (sessions 1 and 3), participants were required to respond according to stimulus color while ignoring its location. Each participant was instructed to respond to only one stimulus color. For half of the pairs, the participant sitting on the right chair was instructed to press the right key to the red stimulus whereas the participant sitting on the left chair was instructed to press the left key to the green stimulus. The other half experienced the opposite mapping. Stimulus-response (S-R) mapping did not change across the two sessions. Color and location of the stimulus varied randomly, but both colors and locations appeared equally often across the experiment. For each participant, two types of trials were included in the experiment: corresponding trials, in which the stimulus appeared on the same side of the screen as the side of the correct response, and non-corresponding trials, in which the stimulus appeared on the side opposite to that of the response.

As in the joint condition of Atmaca et al. [33], during the joint flanker task, participants were presented with an array of five letters and were instructed to respond to the central letter (the target). Targets were the letters H, K, S, and C, with H and K assigned to one response key, and S and C assigned to the other response key. The letters H, K, S, C, and U served as flankers. The combination of target and flanker letters resulted in four stimulus types: identical, compatible, incompatible, and neutral. In identical trials, targets were surrounded by distracting flankers that were identical to the target (e.g., HHHHH); in compatible trials, the flankers differed from the target but referred to the same response (e.g., KKHKK); in incompatible trials, the flankers differed from the target and referred to the opposite response (e.g., SSHSS); finally in the neutral trials, flankers differed from the target and did not refer to any response (e.g., UUHUU). Each participant in the pair was instructed to respond to two of the four target letters (H, K vs. C, S) by pressing the response key on his/her side (left or right key). For instance, the participant sitting on the right was required to respond if the central letter was “ H” or “K” by pressing the right key, and to refrain from responding if the central letter was “C” or “S”. The combinations of target pairs (H, K vs. S, C) and response keys (left vs. right) were counterbalanced across participants. Each participant responded to the same target pair with the same key throughout the experiment.

Following Iani et al. [14], in the cooperative condition, each pair of participants was placed in competition against the other pairs. Participants in the pair were told that the best-performing pair, in terms of both speed and accuracy, would receive a ten Euro reward (five Euro to each participant). In the competitive condition, participants in the pair were placed in a competition against one another. They were told that, at the end of the experiment, the best-performing participant, in terms of both speed and accuracy, would receive a five Euro reward.

In both tasks, a trial began with the presentation of the fixation cross at the center of a black background. After 1000 ms the stimulus appeared. In the Simon task, the stimulus appeared to the right or to the left of the fixation cross and remained visible for 800 ms. Maximum time allowed for a response was 1000 ms. In the Eriksen flanker task, the stimulus appeared in the center of the screen and remained visible for 600 ms. Maximum time allowed for a response was 1000 ms. In both tasks, a response terminated the trial and the inter-trial-interval was 600 ms.

The Simon task consisted of 12 practice trials and 160 experimental trials, divided into two blocks of 80 trials each. The Eriksen flanker task consisted of 24 practice trials and 288 experimental trials, divided into three blocks of 96 trials each.

At the end of both the second and third sessions, participants were required to rate the experimental session using a 7-point bipolar semantic differential scale on the following dimensions: easy - difficult (1 = easy, 7 = difficult), pleasant – unpleasant (1 = pleasant, 7 = unpleasant), positive – negative (1 = positive, 7 = negative), and cooperative–competitive (1 = cooperative, 7 = competitive).

### Results

#### Simon task

**Figure 1:**
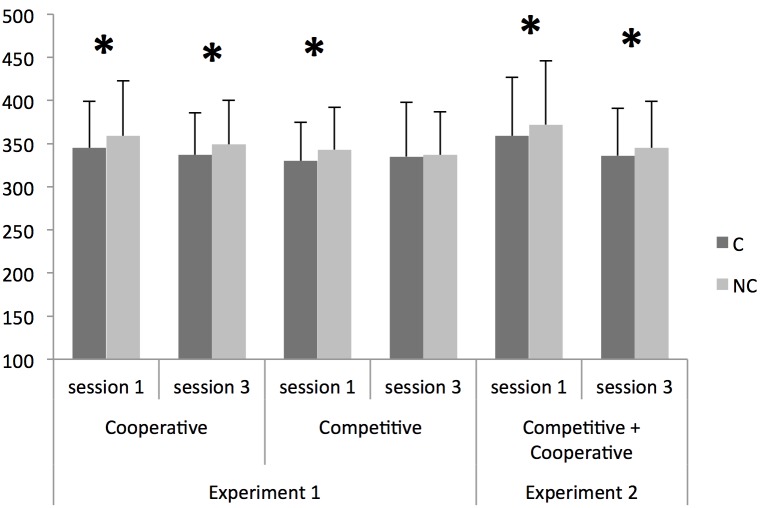
Mean reaction times (±SD) in ms for corresponding and non-corresponding trials for the two conditions of Experiment 1 (cooperative- and competitive-instruction conditions) and for Experiment 2 as a function of session (1 and 3). Asterisks denote significant differences (p<0.05).

Error trials constituted less than 1% of total trials and were not further analyzed. Correct response times (RTs) were submitted to a repeated-measures ANOVA with *Instruction* (cooperative vs. competitive) as between-participants factor, and *Session* (1 vs. 3) and *Correspondence* (corresponding vs. non-corresponding trials) as within-participant factors. Following significant interactions, post-hoc comparisons were performed using the Bonferroni correction. The respective data are shown in Figure 1.

RTs did not differ between the two instruction conditions (348 and 336 ms for the cooperative- and competitive-instruction conditions, respectively), *F*(1,30) = 1.71, *MSe* = 2306, *p* = 0.20, *η_p_^2^* = 0.05. Overall, participants were faster in corresponding (337 ms) than in non-corresponding (347 ms) trials, as indicated by the main effect of *Correspondence*, *F*(1,30) = 15.03, *MSe* = 233, *p*<0.001, *η_p_^2^* = 0.33. Both the two-way interaction between *Session* and *Correspondence*, *F*(1,30) = 10.96, *MSe* = 30.27, *p*<0.01, *η_p_^2^* = 0.26, and the three-way interaction between *Instruction, Session* and *Correspondence*, *F*(1,30) = 5.00, *MSe* = 30.27, *p*<0.05, *η_p_^2^* = 0.14, were significant. No other main effect or interaction reached significance, all *Fs*<1.

As regards the two-way interaction, post-hoc comparisons indicated that the difference between corresponding and non-corresponding trials was significant in both sessions (session 1: 337 vs. 352 ms, for corresponding and non-corresponding trials, respectively, *t*(31) = 4.98, *p*_Bonferroni corrected_<0.001, *d* = 0.88; session 3: 336 vs. 343 ms, for corresponding and non-corresponding trials, respectively; *t*(31) = 2.40, *p*_Bonferroni corrected_ = 0.046, *d* = 0.42). To further assess the three-way interaction between *Instruction*, *Session* and *Correspondence*, we computed the difference in RTs between non-corresponding and corresponding trials (i.e., the Simon effect) and submitted it to a repeated-measures ANOVA with *Session* as within-participant factor and *Instruction* as between-participants factor. This analysis showed a significant main effect of Session, *F*(1,30) = 10.96, *MSe* = 60.53, *p*<0.01, *η_p_^2^* = 0.27, with a larger Simon effect in session 1 (14 ms) than in session 3 (7 ms). The interaction between *Session* and *Instruction* was also significant, *F*(1,30) = 5.00, *MSe* = 60.53, *p*<0.05, *η_p_^2^* = 0.14. Post-hoc comparisons showed that in the cooperative-instruction condition, the 14-ms effect evident in Session 1 did not differ from the 12-ms effect evident in Session 3, *t*(15) = 0.97, *p*_Bonferroni corrected_>0.50, *d* = 0.24. In the competitive-instruction condition, the 2-ms effect evident in Session 3 was significantly different from the 13-ms effect evident in Session 1, *t*(15) = 3.33, *p*_Bonferroni corrected_ = 0.01, *d* = 0.83.

#### Flanker task

Error trials constituted 2.23% of total trials and were not further analyzed. Correct reaction times (RTs) were submitted to a repeated-measures analysis of variance (ANOVA) with *Stimulus type* (identical, compatible, neutral and incompatible) as within-participant factor and *Instruction* (cooperative vs. competitive) as between-participants factor. Following significant interactions, post-hoc comparisons were computed using the Bonferroni correction. The respective data are shown in Table 1.

**Table 1:** Mean reaction times (±SD) in ms in the flanker task for the two conditions of Experiment 1 (cooperative- and competitive-instruction conditions) and for Experiment 2 as a function of stimulus type.

	Experiment 1		Experiment 2
*Stimulus type*	*Cooperative instructions*	*Competitive instructions*	*Competitive instructions*
**Identical**	456 (31.6)	421 (28.4)	449 (38.2)
**Compatible**	467 (35.0)	422 (32.5)	454 (39.7)
**Neutral**	476 (33.3)	432 (34.6)	467 (37.3)
**Incompatible**	496 (31.3)	448 (36.4)	493 (35.0)

The analysis showed a significant main effect of *Instruction, F*(1,30) = 14.98, *MSe* = 3888, *p*<0.01, *η_p_^2^* = 0.33, with faster RTs in the competitive-instruction condition (431 ms) than in the cooperative-instruction condition (473 ms). The main effect of *Stimulus type* was also significant, *F*(3,90) = 44.44, *MSe* = 153.37, *p*<0.001, *η_p_^2^* = 0.60. RTs were 438 ms for identical stimuli, 444 ms for compatible stimuli, 454 ms for neutral stimuli and 472 ms for incompatible stimuli. Post-hoc comparisons indicated that RTs for compatible and identical stimuli did not differ, *t*(31) = 1.79, *p*_Bonferroni corrected_ = 0.50, *d* = 0.32, while all other comparisons were significantly different (identical vs. neutral: *t*(31) =  −5.51, *p*_Bonferroni corrected_<0.001, *d* =  −0.97; identical vs. incompatible: *t*(31) =  −10.06, *p*_Bonferroni corrected_<0.001, *d* =  −1.78; compatible vs. neutral: *t*(31) =  −3.45, *p*_Bonferroni corrected_ = 0.01, *d* =  −0.61; compatible vs. incompatible: *t*(31) = −8.13, *p*_Bonferroni corrected_<0.001, *d* =  −1.44; incompatible vs. neutral: *t*(31) = 5.66, *p*_Bonferroni corrected_<0.001, *d* = 1.00). The two-way interaction between *Instruction* and *Stimulus type* did not reach statistical significance, *F*(3,90) = 1.71, *MSe* = 153.37, *p* = 0.17, *η_p_^2^* = 0.05, indicating that the difference between stimulus types was not modulated by the different instructions.

#### Subjective ratings

Participants’ ratings of sessions 2 and 3 are reported in Table 2. A first analysis was conducted to assess whether participants’ judgments were affected by the instruction manipulation. To this end, a *t* test for independent samples was performed on the scores obtained for the flanker task (session 2). This analysis indicated that participants in the competitive-instruction condition judged the flanker task as more pleasant, *t*(30) =  −1.90, *p*<0.04, *d* = −0.67, and less cooperative, *t*(30) = 1.79, *p*<0.04, *d* = 0.64, than participants in the cooperative-instruction condition. No other difference between the judgments of two groups reached statistical significance (all *p*s>0.05).

**Table 2:** Mean (±SD) subjective ratings for the two sessions (2 and 3) of Experiment 1 (cooperative- and competitive-instruction conditions) and of Experiment 2.

		Experiment 1		Experiment 2
		*Cooperative instructions*	*Competitive instructions*	*Competitive+Cooperative instructions*
**Easy-difficult**	***Session 2***	3.87 (1.26)	3.13 (1.31)*	3.12 (1.31)*
	***Session 3***	1.69 (0.95)*	2.06 (1.12)*	2.25 (1.06)*
**Pleasant-unpleasant**	***Session 2***	3.31 (1.08)*	2.56 (1.15)*	3.44 (1.55)*
	***Session 3***	2.31 (1.09)*	2.13 (0.96)*	2.75 (1.06)*
**Positive-Negative**	***Session 2***	2.81 (0.98)*	2.44 (1.46)*	2.81 (1.17)
	***Session 3***	2.06 (0.93)*	1.88 (0.81)*	2.56 (1.15)*
**Cooperative-competitive**	***Session 2***	2.44 (1.15)*	3.37 (1.75)	4.06 (1.53)
	***Session 3***	2.19 (1.17)*	2.44 (1.36)*	1.81 (0.91)*

Asterisks indicate values significantly different from the neutral point (4).

A second analysis was performed to assess whether subjective ratings differed between sessions 2 and 3. To this end, for each experimental group, a paired sample *t* test was performed on the scores obtained in the two sessions. For the participants in the cooperative-instruction condition, session 3 was judged as easier, *t*(15) = 7.49, *p*<0.001, *d* = 1.87, more pleasant, *t*(15) = 3.16, *p*<0.01, *d* = 0.79, and more positive, *t*(15) = 2.82, *p*<0.05, *d* = 0.70, than session 2. Scores in the cooperative-competitive dimension did not differ between sessions (*p* = 0.59). For the participants in the competitive-instruction condition, session 3 was judged as easier than session 2, *t*(15) = 2.79, *p*<0.05, *d* = 0.69, and as more cooperative, *t*(15) = 3.34, *p*<0.01, *d* = 0.83, than session 2. All other scores did not differ (*p*s>0.14).

Finally, a one-sample *t* test was used to assess whether the scores for the two sessions significantly differed from the neutral point (4). For the participants in the cooperative-instruction condition, all judgments were significantly lower than the neutral point (*p*s<0.02, *d*s ranging from −2.43 to −0.64), with the exception of the easy-difficult dimension in session 2 (*p* = 0.70). For the participants in the competitive-instruction condition, only the cooperative–competitive dimension in session 2 obtained a score equal to 4, *t*(15) =  −1,4, *p* = 0.17, *d* =  −0.36, while scores in all other dimensions were lower than 4 (*p*s<0.02, *d*s ranging from −2.63 to −0.66).

### Discussion

Results indicated that competition experienced in one task affected the way the subsequent joint Simon task was performed by eliminating the joint Simon effect. This occurred even though, during the Simon task, participants were not explicitly required to compete. On the contrary, a regular joint Simon effect emerged when in the previous task participants were required to cooperate and it was equivalent in size to the effect observed before the instruction manipulation was introduced (session 1). These findings suggest that the need to compete during performance of a task leads to a carry-over effect on joint performance of a subsequent task. Hence, the carry-over effect of competition described by social psychology studies in intergroup contexts (e.g., [20]) occurs even when competition does not concern groups but dyadic interactions.

Performance on the flanker task was not affected by the instruction manipulation, except for an overall speeding up of RTs in the competitive instruction condition. The finding that, differently from what is observed in the social version of the Simon task, the flanker effect was present even when co-acting individuals were required to compete is not surprising and may be explained by the differences between the two tasks. To explain this point, we refer to the dimensional overlap model proposed by Kornblum, Hasbroucq, and Osman [34], which classifies conflict tasks according to the degree of similarity (overlap) between stimulus and response dimensions. The model proposes that, despite of task instructions, there is a tendency for automatic associations among those stimulus and response dimensions that share a high degree of similarity with one another. In the Simon task conflict arises because of the overlap between the irrelevant stimulus dimension (location) and the response dimension (response conflict). Differently, in the Eriksen flanker task, the irrelevant stimulus dimension overlaps with the relevant stimulus dimension (stimulus conflict). In the go/no-go version of the Simon task, in which participants respond to only half of the target stimuli by pressing a single response key, the Simon effect does not typically emerge (e.g., [11]). In the go/no-go version of the flanker task the conflict between relevant and irrelevant stimulus features is still present. Indeed the effect emerges, even if it appears to be smaller than the effect found when the task is performed jointly with a co-actor (e.g., [33], Experiments 1–2) or in the presence of an attention-capturing object such a Japanese waving cat [35].

Crucially, the results of the subjective ratings suggested that the instruction manipulation was effective: during performance of the joint flanker task (session 2) participants under the competitive-instruction condition perceived the situation as less cooperative as compared to participants under the cooperative-instruction condition.

## Experiment 2

Experiment 2 was aimed at assessing whether the introduction of a common goal requiring participants to cooperate can neutralize the carry-over effect of competition observed in Experiment 1. To this end, participants were required to perform a joint Simon task under neutral instructions (session 1), then to perform a joint flanker task under competitive instructions (session 2), and finally to perform a joint Simon task under cooperative instructions (session 3). This procedure can allow us to assess whether, as found in intergroup contexts (e.g., [23]), the carry-over effect of competition found in Experiment 1 can be contrasted by placing participants in a cooperative setting after performing in a competitive one. If this is the case, the joint Simon effect should be evident in both sessions 1 and 3.

### Methods

#### Ethics statement

The study was conducted in accordance with the Declaration of Helsinki and all procedures were approved by the Department of Communication and Economics of the University of Modena and Reggio Emilia.

#### Participants

Sixteen new undergraduate students (13 females, age range: 20–27 years), selected as in Experiment 1, took part in Experiment 2. All gave their written consent. All were naive with regard to the hypotheses of the experiment and were debriefed about the experimental aims at the end of the experiment.

#### Apparatus, stimuli and procedure

Apparatus, stimuli, and procedure were the same as in Experiment 1 except for what follows. During the joint flanker task (session 2), participants were required to compete one against the other. During the following joint Simon task (session 3) they were explicitly required to cooperate to win a ten Euro reward (five Euro to each participant) as best performing pair.

### Results and Discussion

#### Simon task

Error trials constituted less than 1% of total trials and were not further analyzed. Correct RTs were submitted to a repeated-measures ANOVA with *Session* (1 vs. 3) and *Correspondence* (corresponding vs. non-corresponding trials) as within-participant factors. Following a significant interaction, post-hoc comparisons were computed using the Bonferroni correction. The respective data are shown in Figure 1.

The main effects of *Session*, *F*(1,15) = 14.32, *MSe* = 704, *p*<0.01, *η_p_^2^* = 0.49, and *Correspondence*, *F(*1,15) = 16.47, *MSe* = 115, *p*<0.01, *η_p_^2^* = 0.52, were significant. Participants were faster in session 3 (341 ms) than in session 1 (366 ms), and in corresponding (348 ms) than in non-corresponding (359 ms) trials. The interaction between *Correspondence* and *Session* did not reach statistical significance, *F*(1,15) = 3.07, *MSe* = 22, *p* = 0.10, *η_p_^2^* = 0.17. RTs for corresponding trials were 359 ms in session 1 and 336 ms in session 3, while RTs for non-corresponding trials were 372 ms in session 1 and 345 in session 3. Given the *p* and *η_p_^2^* values for the interaction, we decided to perform post-hoc comparisons. These tests confirmed that the difference between corresponding and non-corresponding trials was significant in both sessions (*t*(15) = 4.41, *p*_ Bonferroni corrected_ = 0.002, *d* = 1.10 for session 1; *t*(15) = 3.02, *p*_Bonferroni corrected_ = 0.018, *d* = 0.75 for session 2).

#### Flanker task

Error trials constituted 3.04% of the total trials and were not further analyzed. Correct RTs were submitted to a repeated measure ANOVA with *Stimulus type* as within-participant factor. The respective data are shown in Table 1.

The main effect of *Stimulus type* was significant, *F*(3,45) = 34.16, *MSe* = 183.39, *p*<0.001, *η_p_^2^* = 0.69. RTs were 449 ms for identical stimuli, 454 ms for compatible stimuli, 467 ms for neutral stimuli and 493 ms for incompatible stimuli. Post-hoc comparisons showed that RTs for compatible and identical stimuli did not differ, *t*(15) = 1.07, *p*_Bonferroni corrected_>0.50, *d* = 0.27. All other comparisons significantly differed (identical vs. neutral: *t*(15) =  −4.33, *p*_Bonferroni corrected_ = 0.006, *d* =  −1.08; identical vs. incompatible: *t*(15) =  −8.09, *p*_Bonferroni corrected_<0.001, *d* =  −2.02; compatible vs. neutral: *t*(15) =  −3.01, *p*_Bonferroni corrected_ = 0.05, *d* = −0.75; compatible vs. incompatible: *t*(15) =  −8.84, *p*_Bonferroni corrected_<0.001, *d* =  −2.21; incompatible vs. neutral: *t*(15) = 4.84, *p*_Bonferroni corrected_<0.001, *d* = 1.21).

#### Subjective ratings

Participants’ ratings of sessions 2 and 3 are reported in Table 2. A paired sample *t* test was used to assess whether the scores differed between sessions 2 and 3. Participants judged session 3 as easier, *t*(15) = 2.90, *p*<0.05, *d* = 0.73, more pleasant, *t*(15) = 2.71, *p*<0.05, *d* = 0.68, and more cooperative, *t*(15) = 5.73, *p*<0.001, *d* = 1.43, than session 2. The positive-negative dimension did not differ (*p* = 0.26). A one-sample *t* test showed that in session 2, scores were significantly lower than the neutral point (4) in the easy-difficult dimension, *t*(15) =  −2,67, *p*<0.05, *d* = −0.66, and in the positive-negative dimension, *t*(15) = −4.069, *p*<0.01, *d* =  −1.03. The other scores did not differ from 4 (*p*s>0.17). In session 3, all scores were significantly lower than the neutral point (*p*s<0.01, *d*s ranging from –2.41 to −1.18). Again, these results confirm that the instruction manipulation was effective: Session 2 was perceived as more competitive than Session 3.

While in Experiment 1 we found that competition experienced in one task affected the way a subsequent joint task with no explicit competitive or cooperative instructions was performed, in the present experiment we found that a significant joint Simon effect emerged when participants were placed in a cooperative condition after performing a task under competitive instructions. This evidence suggests that the carry-over effect of competition found in Experiment 1 can be neutralized by the introduction of a common goal that cannot be achieved if the two individuals do not cooperate. This finding is in line with the results by Sherif et al. [23] found in group settings and shows that such results can be applied to dyadic interactions as well.

## General Discussion

Previous results showed that the joint Simon effect does not emerge when co-actors need to compete one against the other [14]. The aim of the present study was twofold. First, we investigated whether the influence of competition experienced in a specific joint action context extends to a following unrelated joint task in which competition is not explicitly required. In addition, we assessed whether this influence vanishes when, following competition, participants are required to cooperate.

In Experiment 1 we asked participants to perform a joint Simon task with neutral instructions before and after performing a joint non-spatial task in which they were explicitly required either to cooperate with or to compete against a co-actor. Results demonstrated that cooperation experienced during the non-spatial task did not affect performance on the subsequent joint Simon task: a significant joint Simon effect was evident in session 3 and its magnitude was comparable in size to the effect found in session 1. This result suggests that the need to cooperate does not further increase the entity of the joint Simon effect. Hence, it seems that, in the absence of specific instructions, individuals acting in a social context may by default choose to cooperate (see also [14]). This finding is consistent with the view that humans are inclined to share goals and intentions, and to cooperate with the others (e.g., [36], [37]).

Crucially, the introduction of competing goals disrupted this tendency. Indeed, the joint Simon effect was absent when during the preceding task participants were instructed to compete one against the other. Even though the instruction manipulation did not affect flanker task performance except for an overall speeding up of response times under the competitive instructions, it influenced how the task situation was perceived. Indeed, participants in the competitive instruction condition perceived the task situation as less cooperative as compared to participants in the cooperative instruction condition. Hence, these results suggest that competition experienced in one task transferred to a following unrelated task, even though during the latter participants were no longer required to compete, thus preventing the emergence of the joint Simon effect. This result is particularly relevant because it demonstrates that competition experienced in one task may have carry-over effects on subsequent unrelated tasks and that these effects may be strong enough to contrast the natural tendency to cooperate. As suggested by Sassenberg et al. [20], the concept of mindset may provide a useful framework to explain these carry-over effects. Empirical evidence suggests that competitive and cooperative interactions are characterized by different neural activations [18], involve different motor planning (e.g., [38]), and influence which and how visual information about the others is used (e.g., [39]). On a more general level, it can be argued that cooperation and competition lead to the activation of different mindsets, that is, mental mechanisms functional to attain specific goals. Once a specific mindset has been activated to deal with a specific task, it tends to be applied to following situations unless a change in situational demands makes a change in the current mindset necessary.

Experiment 2 allowed us to assess whether the carry-over effect of competition can be neutralized when participants, after competing one against the other in one task, are explicitly required to cooperate during a subsequent unrelated task. Our results indicated that competition experienced in one task no longer affected subsequent performance when a new cooperative goal (i.e., a goal that created positive interdependence between the two agents) was introduced. This finding is consistent with the results by Sherif et al. [23] showing that, in an intergroup context, the negative effects deriving from the need to compete vanish only when a superordinate goal is introduced.

To note, the authors embracing the idea that the joint Simon effect reflects task/action co-representation have originally suggested that individuals automatically integrate self and other’s action in a shared representation any time they act in a social context (e.g., [1], [40]). Neurophysiological data showing that individuals need to engage in inhibitory control processes to withhold from responding when is the other’s turn (e.g., [41]) have been taken as evidence of this. The automatic nature of shared representations, however, has been put into question by the results of a series of recent studies suggesting that emotional [31] and social (e.g., [14], [42]) factors as well as the style of thinking [43] may modulate their emergence. The results of the present study are in line with these latter studies since they show that the emergence of the joint Simon effect is strongly influenced by whether the goals of the co-acting individuals are positively (as in cooperative settings) or negatively (as in competitive settings) interdependent.

It is important to consider that not all researchers agree with the purely social interpretation of the joint Simon effect originally proposed by Sebanz et al. [1]. There are indeed recent findings suggesting that the joint Simon effect might be due to spatial factors (e.g., [11], [12], [44]). For instance, Dolk et al. [11] recently suggested that the joint Simon effect might arise because participants code their own actions with respect to salient action events in order to discriminate between them. According to their view, the degree of similarity between one’s own actions and other salient action events determines whether this discrimination is necessary. If the co-actors perceived themselves as different, there is no need to spatially code own and other’s actions. The present findings and those by Iani et al. [14] showing that the joint Simon effect does not emerge when co-actors are required to compete one against the other could be easily reconciled with this view. Indeed, it could be hypothesized that the joint Simon effect did not emerge in the competitive condition because competition increased the perceived differences between co-actors (e.g., [17]), thus rendering the spatial coding of actions unnecessary.

To conclude, although the results of the present study do not allow us to disentangle among the different interpretations of the joint Simon effect, they clearly demonstrate that the influence of competition may extend beyond the specific setting in which it is experienced and it is so strong to affect subsequent interactions.
